# The Duration of Auditory Sensory Memory for Vowel Processing: Neurophysiological and Behavioral Measures

**DOI:** 10.3389/fpsyg.2018.00335

**Published:** 2018-03-22

**Authors:** Yan H. Yu, Valerie L. Shafer, Elyse S. Sussman

**Affiliations:** ^1^Department of Communication Sciences and Disorders, St. John’s University, Queens, NY, United States; ^2^Ph.D. Program in Speech-Language-Hearing Sciences, The Graduate Center, The City University of New York, New York, NY, United States; ^3^Dominick P. Purpura Department of Neuroscience, Albert Einstein College of Medicine, New York, NY, United States

**Keywords:** sensory memory decay, vowel processing, mismatch negativity, late negativity, event-related potentials, speech perception, interstimulus interval, P3a novelty

## Abstract

Speech perception behavioral research suggests that rates of sensory memory decay are dependent on stimulus properties at more than one level (e.g., acoustic level, phonemic level). The neurophysiology of sensory memory decay rate has rarely been examined in the context of speech processing. In a lexical tone study, we showed that long-term memory representation of lexical tone slows the decay rate of sensory memory for these tones. Here, we tested the hypothesis that long-term memory representation of vowels slows the rate of auditory sensory memory decay in a similar way to that of lexical tone. Event-related potential (ERP) responses were recorded to Mandarin non-words contrasting the vowels /i/ vs. /u/ and /y/ vs. /u/ from first-language (L1) Mandarin and L1 American English participants under short and long interstimulus interval (ISI) conditions (short ISI: an average of 575 ms, long ISI: an average of 2675 ms). Results revealed poorer discrimination of the vowel contrasts for English listeners than Mandarin listeners, but with different patterns for behavioral perception and neural discrimination. As predicted, English listeners showed the poorest discrimination and identification for the vowel contrast /y/ vs. /u/, and poorer performance in the long ISI condition. In contrast to Yu et al. (2017), however, we found no effect of ISI reflected in the neural responses, specifically the mismatch negativity (MMN), P3a and late negativity ERP amplitudes. We did see a language group effect, with Mandarin listeners generally showing larger MMN and English listeners showing larger P3a. The behavioral results revealed that native language experience plays a role in echoic sensory memory trace maintenance, but the failure to find an effect of ISI on the ERP results suggests that vowel and lexical tone memory traces decay at different rates.

**Highlights**:

We examined the interaction between auditory sensory memory decay and language experience.

We compared MMN, P3a, LN and behavioral responses in short vs. long interstimulus intervals.

We found that different from lexical tone contrast, MMN, P3a, and LN changes to vowel contrasts are not influenced by lengthening the ISI to 2.6 s.

We also found that the English listeners discriminated the non-native vowel contrast with lower accuracy under the long ISI condition.

## Introduction

Mismatch negativity (MMN) is an event-related brain potential (ERP) component that is elicited by a stimulus that is detected as a violation of automatic predictions of the central auditory system (Näätänen et al., 2011). MMN was traditionally interpreted in terms of echoic auditory sensory memory, which can last ca. 10 s as a result of repeating standard stimuli in the central auditory system (e.g., Näätänen et al., 1978; Cowan, 1984, 1988). The strength and durability of the echoic sensory memory trace, as reflected by the MMN, is affected by a number of factors, including the number of standard stimulus repetitions before the deviant (Sams et al., 1983; Imada et al., 1993; Javitt et al., 1998), the acoustic distinctiveness of the standard-deviant contrast (e.g., Sams et al., 1985), the linguistic status of the contrast (e.g., Näätänen et al., 1997), and the rate of stimulus presentation (e.g., Schröger, 1996; Sussman et al., 2008). More specifically, smaller MMN amplitude has been observed to a smaller magnitude of stimulus change in tone frequency, duration or intensity (e.g., Sams et al., 1985; Lang et al., 1990; Tiitinen et al., 1994; Amenedo and Escera, 2000; Rinne et al., 2006). Studies have also shown smaller or absent MMNs for non-native compared to native phonetic contrasts that are phonemic only for the native group (Dehaene-Lambertz, 1997; Näätänen et al., 1997; Sharma and Dorman, 1999; Winkler et al., 1999a). MMN also decreases in amplitude when the ISI between standard and deviant auditory stimuli is increased (Sams et al., 1993; Schröger, 1996; Čeponienė et al., 1998, 1999; Javitt et al., 1998; Gomes et al., 1999; Barry et al., 2008). Horváth et al. (2008a) argued that the amplitude of MMN varies little in the magnitude from trial to trial for a clearly discriminable difference; in contrast the smaller MMN amplitude to a just-noticeable difference may reflect that deviance detection occurs only for a subset of the trials.

Sensory memory decays in a non-linear fashion, but this decay rate is dependent on a number of factors. Behavioral speech perception research suggests that the rate of sensory memory decay is dependent on stimulus properties at more than one level. For example, at the acoustic level, a “simpler” (steady-state) vowel has an advantage over a “complex” (brief and transitional) consonant in terms of the rate of decay (Pisoni, 1973). At a phonemic level, a between-category but not with-category consonant contrast that differs equally on an acoustic scale, can be retained for successful behavioral discrimination at a minimal ISI of 1.5 s, and possibly at a much longer ISI than 1.5 s. For example, Hindi participants had no difficulties discriminating the between-category Hindi sounds as different at an ISI of 1.5 s (Werker and Logan, 1985).

### Mismatch Negativity and ISI Modulation

The interactions between sensory memory trace decay and auditory discrimination, as measured by the MMN responses, have been investigated using pure tones (e.g., Sams et al., 1993), consonant contrast (Čeponienė et al., 1999) and lexical tones (Yu et al., 2017). The accumulated evidence thus far suggests that the rate of sensory memory trace decay may differ depending on the nature of the stimulus. For example, Sams et al. (1993) found that in healthy young adults, MMN can be elicited with an ISI as long as 10 s when the stimuli are auditory pure tones that differ in frequency by 10%. Čeponienė et al. (1999) found that two groups of 7–9 years old children with high and low phonological memory skills, as measured by non-word repetition (NWR), showed very similar MMN responses to auditory tone changes (1000 Hz vs. 1100 Hz) under both short (350 ms) and long (2000 ms) ISI conditions. However, the higher NWR repeaters differed from the lower NWR repeaters in terms of the MMN amplitude for a consonantal voicing contrast (/baga/-/baka/). The MMN amplitude was greatly reduced in high repeaters under the long ISI condition compared to that of the short ISI condition, and no MMN was observed for either the short or long ISI conditions in the low repeaters. In Yu et al. (2017), we found that native speakers of English failed to show an early negativity (i.e., MMN) to a Mandarin lexical tone contrast, which is phonemic in Mandarin but not English, when the ISI was greater than 2.5 s; however, English listeners did show MMN to the lexical tone contrast under a short ISI of approximately 500 ms. The Mandarin native listeners showed comparable MMNs for both the long and short ISI.

To our knowledge, there is no study that has directly examined the duration of the neuronal trace of auditory sensory memory for vowels. Recent evidence suggested that consonants, vowels and lexical tones are weighted differently (Wiener and Turnbull, 2016). In this paper, we examined whether the rate of sensory memory decay for vowels differs from the rate of decay for lexical tone reported in Yu et al. (2017).

### P3a and ISI Modulation

The P3a component (sometimes called novelty P3), a frontal-central positivity, is often considered to be a correlate of involuntary attention switch in a passive listening paradigm (e.g., Squires et al., 1975; Polich, 1988, 2007; Escera et al., 1998; Friedman et al., 2001 for reviews). Both the magnitude of stimulus deviance and the probability of the deviant occurrence affect the peak amplitude of P3a. Larger magnitude of stimulus deviance and lower probability of the deviant lead to a larger P3a peak amplitude (e.g., Schröger and Wolff, 1998a,b; Winkler et al., 1998; Escera et al., 2001; Yago et al., 2001). Sussman et al. (2003) found that P3a was present when the deviant was unpredictable and absent when the deviant was predictable, suggesting that P3a represents an involuntary orienting of attention to an unexpected sound (Sussman et al., 2003). Larger P3a amplitudes evoked by non-native contrasts compared to native contrasts have been observed (e.g., Shestakova et al., 2003) and late bilingual learners have larger P3a amplitudes than early bilingual learners (Ortiz-Mantilla et al., 2010). Few studies have examined how ISI modulates the P3a responses. The question of whether P3a is sensitive to ISI modulation has yet to be established. In a recent systematic review by Bartha-Doering et al. (2015), out of 37 MMN studies that have ISI manipulation, there was only one study that also discussed P3a responses (Czigler et al., 1992). Czigler et al. (1992) reported an unexpected P3a component in young adults (age = 21.3 years, *n* = 8) when the ISIs were 800 and 2400 ms, but the P3a was absent in the same participants when the ISI was longer (up to 7200 ms). Furthermore, no P3a was observed in healthy older adults (age = 60.8, *N* = 8).

### Late Negativity and ISI Modulation

In a passive oddball paradigm, following MMN and P3a, a frontocentral late negativity (LN) is often generated. The LN is considered an index of re-orienting to the stimulus change, similar to the re-orienting negativity (RON) reported in Schröger and Wolff (1998a) (see Escera et al., 2000 for a review). The LN has been less frequently reported, but an increasing number of studies suggest that it is a fairly common mismatch response (Korpilahti et al., 2001; Shestakova et al., 2003; Shafer et al., 2005; Kaan et al., 2007; Barry et al., 2009; Bishop et al., 2010; Datta et al., 2010; Ortiz-Mantilla et al., 2010; Choudhury et al., 2015). The question of how language experience modulates LN responses has not been systematically explored. Some studies reported larger LN amplitude in higher language performers (e.g., Barry et al., 2009), while others reported the opposite results. Several studies observed that LN is larger to speech contrasts in second language learners (e.g., Ortiz-Mantilla et al., 2010), or in other clinical groups (e.g., Addis et al., 2010). Shafer et al. (2005) observed an LN of comparable amplitude in children with specific language impairment (SLI) and age-matched controls with typical language.

Late negativity amplitude and latency are also found to interact with the salience of an acoustic contrast, the rate of presentation and with attention (e.g., Choudhury et al., 2015). Choudhury et al. (2015) have found that attending to a very fast-rate stimulus contrast (an ISI of 10 or 70 ms) led to larger LN amplitude compared to a slow-rate stimulus. In the lexical tone study, we found larger LN amplitudes in the native Mandarin listeners than in the English listeners for Mandarin lexical tone contrasts (Yu et al., 2017). The amplitude of LN in the Mandarin listeners was either the same or larger in the long ISI condition compared to the short ISI condition. It currently is unclear whether LN is actually a second MMN, or rather a separate component. In addition, very few studies have examined how memory trace decay affects LN (Yu et al., 2017). Thus, further research is necessary to elucidate the nature of the LN, and how ISI modulates the LN responses.

### The Role of Long-Term Memory in Non-native Speech Processing

For the purpose of this study, we define long-term memory as the established mental representation of phonemic categories of native language experience. It is well established that the amplitude of MMN to native language phonemic contrasts is larger than for non-native speech contrasts that are non-phonemic in the listener’s native language (Dehaene-Lambertz, 1997; Näätänen et al., 1997; Sharma and Dorman, 1999; Winkler et al., 1999a). Speech perception models, such as the perceptual assimilation model (PAM) posited that the first language (L1) system constrains the perception of non-native speech sounds that are unfamiliar to the listener (Best, 1995; Best and Tyler, 2007). A postulate of PAM is that naïve listeners perceptually assimilate a non-native phone to the native phoneme that is the closest articulatory match, if the listeners can find a match (good or poor) in their L1 (Best and Tyler, 2007).

Mandarin has six primary vowels (excluding diphthongs). These include high-front /i/ and high-back /u/ vowels that are similar to English (Howie, 1976; Lee and Zee, 2003) and high, front-rounded vowel /y/. English, in contrast, has more vowel distinctions (11 excluding diphthongs), but does not include the front-rounded vowel /y/. Furthermore, English includes the constraint on back vowels that they are all round (+round feature), whereas front vowels are non-round (-round). Thus, PAM predicts that English listeners will assimilate the Mandarin /y/ into the most similar category, and given the constraint on English front vowels lacking the feature “round,” English /u/ (high, and round) should be closest match.

The one study that has tested behavioral discrimination of Mandarin /y/ vs. /u/ by naïve English speaker revealed that the difference can be discriminated well-above-chance (mean 92% correct) (Hao, 2017). This study used an AXB task (with 1 s between three-syllable phrases) rather than identification and did not compare performance to Mandarin listeners. In a different study examining identification of the French front-rounded /y/ vs. back-rounded /u/, English listeners categorized /y/ in a single category with /u/ (Levy and Strange, 2008).

According to PAM and the findings regarding French /y/, English listeners are expected to show poorer discrimination of Mandarin /y/ and /u/ than Mandarin listeners, assimilating these into one category [called “single-category (SC) assimilation in PAM]. The Hao (2017) study, however, suggests that English listeners will show evidence of good discrimination. With regards to Mandarin /u/ vs. /i/, English listeners are expected to assimilate these into two separate categories [called two category (TC) assimilation in PAM] that correspond to English /u/ and English /i/. Thus, higher behavioral discrimination and identification accuracy is expected for /u/ vs. /i/ than for Mandarin /y/ vs. /u/.

### Objectives of This Study

We investigated whether the interstimulus rate (ISI) influenced neural responses to infrequent vowel changes and to what extent this was modulated by language experience. A second aim was to determine whether vowel processing and lexical tone processing differ at the neural level in terms of sensory memory decay. Answers to these questions are of interest for theoretical, as well as for practical reasons. With regards to language experience, it is important to know whether different phonological properties (vowel, consonant, and lexical tone) show different decay rates in relation to experience. In addition, these results will allow us to estimate the potential of using sensory memory decay as a reliable means for assessing implicit cognitive information-processing capabilities in applied/clinical testing situations. A recent systematic review of how ISI modulates the amplitude of MMN revealed that factors, such as maturation and normal aging influence the duration of sensory memory (Bartha-Doering et al., 2015). However, the studies reviewed in Bartha-Doering et al. (2015) are oddball paradigms using pure tones as stimuli. Few studies have directly compared the rate of sensory memory decay for different types of linguistic stimuli (e.g., vowel, consonant, and lexical tone) (e.g., consonant: Čeponienė et al., 1999; lexical tone: Yu et al., 2017). The question remains open as to whether the type of stimuli interact with the rate of sensory memory decay. This study focuses on vowel contrasts, which we can then compare to our previous study of lexical tone (Yu et al., 2017).

The current study focuses on the modulation of MMN, P3a and LN under different ISI conditions for two vowel contrasts, one of which is phonemic (/i/–/u/) for both English and Mandarin listeners, and a second (/y/–/u/) which is phonemic only for Mandarin listeners. We used an average ISI of 575 ms (range 545–609 ms) for the short ISI condition and an average ISI of 2675 ms, (range of 2645–2709 ms) for the long ISI condition (see Yu et al., 2017 for details). We hypothesized that native language experience would interact with the rate of memory trace decay. The two language groups would show larger dissimilarity in the behavioral and ERP responses for the long ISI compared to the short ISI. This is because when the ISI is short, listeners can rely on the acoustic-phonetic cues for discrimination of non-native speech contrast while with a long ISI, the detailed acoustic-phonetic cues would not be available, and speech processing has to rely on the long-term memory representation. Thus, behavioral perception and the MMN brain discriminative response would be poorer at the long vs. the short ISIs and poorer for English than Mandarin listeners for the /y/ vs. /u/ contrast.

## Materials and Methods

### Participants

We tested 68 adult participants between the age of 20 and 42 years of age using a between-subject design. Data from a total of five participants (two from the English short ISI group, two from the Mandarin short ISI group, and one from the Mandarin long ISI group) were excluded from the analysis due to incomplete participation (*N* = 2), or excessive noise in the data defined by retaining less than 50% of trials after artifact reject (*N* = 2) or no clear obligatory components (*N* = 1). Therefore, a total of 63 adult participants were included in the analyses. There were 15 participants in the English long ISI group (8 males, 7 females), 16 participants in the English short ISI group (7 males, 9 females), 16 (9 males, 7 females) in the Mandarin long ISI group and 16 (8 males, 8 females) in the Mandarin short ISI group. The 31 native speakers of English (16 in the short ISI and 15 in the long ISI condition, age range: 20–42 years) had little or no exposure to any tone languages. The 32 native speakers of Mandarin (16 participants in each ISI condition, age range: 21–40 years) were all from Mainland China, and all moved to the United States no earlier than during their high school years. Participants’ age, gender, music training and handedness were controlled across language/ISI groups. These were the same participants as in Yu et al. (2017). The study was approved by The Graduate Center, CUNY Institutional Review Board, and was conducted in compliance with the *Declaration of Helsinki*. All the participants signed the informed consent. All the participants were recruited from the metropolitan New York City area via flyers and Craigslist, and were paid $10 per hour.

### Stimuli

The stimuli were produced in Mandarin by a bilingual Mandarin-English female speaker, for whom Mandarin was the first language (lived in China until 20 years of age). They were recorded using Sound Forge 4.5 at the sampling rate of 22,050 Hz. A total of 11 tokens of stimuli were selected from a large set of stimuli after batch normalization using average RMS normalization function in Sound Forge 8.1 and detailed acoustic analyses using Praat (Boersma, 2001). Among these 11 tokens, three were for the standard “gupa” condition (with “gu” in Mandarin Tone 3, a low rising fundamental frequency contour) and two were for each of the four deviant stimulus types. The two vowel deviants were “gipa” and “gypa”, both with Tone 3 on the first syllables, and the two lexical tone deviants were “gupa” with a rising tone (Tone 2) on the first syllable, and “gupa” with a high level tone (Tone 1) on the first syllable. The second syllable “pa” was always produced with Tone 1 (see **Table 1** for acoustic details). To verify the validity of the stimuli, before we launched the experiment, four native Mandarin adult listeners (one Ph.D. student in speech and hearing, two journalists, and one manager) judged that all the stimuli were native-sounding Mandarin speech. The rationale for using more complex stimuli and multiple deviant conditions was to increase the ecological validity of the task and to enhance the possibility of participants relying on phonological processing instead of phonetic processing. In this paper, we focus on the vowel differences.

**Table 1:** Characteristics of the experimental stimuli.

Stimuli	/gipa/		/gypa/		/gupa/		
	Token 1	Token 2	Token 1	Token 2	Token 1	Token 2	Token 3
F0-gV (Hz)	153	155	138	142	140	142	143
F0-pa (Hz)	174	173	166	185	167	168	171

Duration:overall (ms)	314	336	342	355	320	326	346
Voice onset time /g/	34	24	16	36	21	22	23
Duration:gV(ms)	114	113	129	157	132	139	134
Duration: pa (ms)	200	223	213	198	188	187	212

Intensity:overall (dB)	71.4	67.9	66.8	68.1	68.8	70.9	70.8
Intensity:gV (dB)	70.8	72.1	62.6	66.9	69.3	70.6	71.6
Intensity:pa (dB)	71.8	66	68.8	68.9	68.4	71.1	70.4

**Formant Frequency**							
F1:gV (Hz)	330	328	306	327	345	345	341
F2:gV (Hz)	2437	2298	1626	1792	1013	1102	1154
F3:gV (Hz)	2980	2635	2360	2330	2622	2630	2688
F1:pa (Hz)	686	691	659	715	722	766	778
F2:pa (Hz)	1601	1544	1595	1399	1538	1465	1478
F3:pa (Hz)	3065	2912	3062	2999	2681	2648	2730

*“gV” stands for the first syllable, and “pa” stands for the second syllable of the stimuli. ERP responses from two tokens of /gipa/ and two tokens of /gypa/ and three tokens of /gupa/ were included in the analyses*.

### Paradigm

Participants were seated in a sound-attenuated and electrically shielded booth for a passive listening MMN paradigm and behavioral tasks. E-Prime software (Psychology Software Tools, Pittsburgh, PA, United States) was used for stimulus presentation and behavioral data collection. A 65-channel Geodesic sensor net was used for ERP data collection.

**FIGURE 1:**
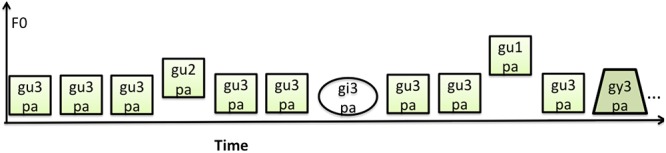
Schematic of the experimental paradigm.

One block consisted of 103 pseudo-randomly presented stimuli, and a total of twenty blocks were presented with an interblock interval of 20 s. The vowel and lexical tone deviant stimuli were interspersed within the sound stream of common standard stimulus type /gu3pa/. **Figure 1** provides examples of the stimulus sequences used in the study. A total of 200 deviant trials per category with a probability of 9.7% and a total of 1260 standard stimuli with a probability of 62.2% were presented. The standard stimuli that were at the beginning of the list, or following a deviant of any type, were excluded from the analyses. A total of 440 trials of standard stimuli were included in the offline ERP analyses. The /gupa/ with a different tone were not included in the analysis as they had a dual role of serving as the vowel standard and lexical tone deviant in the experiment.

A behavioral discrimination task for the vowels (four standard and a fifth/final deviant) followed the ERP measurement. No EEG activity was recorded during the behavioral testing. Participants were asked to determine whether the stimulus in the final position of the sequence of five stimuli was the same or different from the previous four stimuli. The ISI between stimuli was the same as for the ERP paradigm, that is, short ISI for participants receiving the short ISI ERP condition and long ISI for those receiving the long ISI ERP condition. A three-alternative forced choice identification task with “gipa,” “gupa,” or “gypa” as the alternatives was presented last. Each participant received six practice trials plus 30 test trials, and each was asked to press button “1” for “gipa,” button “2” for “gupa,” and button “3” for “gypa.” The purpose of this task was to determine the category perception.

We also included a post-study test to evaluate how well each vowel stimulus matched the English /i/ and /u/ categories. Fourteen native English undergraduate students were asked to report which vowel they heard in the first syllable of “gVpa,” then using a 5-point Likert scale to judge how English-sounding each token of “gVpa” was (“1” = native English sounding, “3” somewhat English sounding, “5” = not English sounding at all). Nine out of 14 listeners identified the two tokens of “gipa” as English “gipa,” and 10 of 14 listeners identified the three tokens of “gupa” as English “gupa.” Twelve out of 14 listeners judged the first token of “gypa” as “gupa,” and all 14 listeners judged the second token of “gypa” as “gupa.” The English-sounding ratings for the two tokens of “gipa” were 2.6 and 2.8. The three tokens of “gupa” received the ratings of 2.9, 3.0, and 3.2, and the two tokens of “gypa” were rated as 3.56 and 3.50. Thus, the majority of the listeners consistently identified the Mandarin /i/ and /u/ tokens as best matching with English /i/ and /u/, respectively, whereas both Mandarin /y/ tokens were placed with English /u/.

All stimuli were presented free-field with a comfortable listening level of 70.2 dB (*SD* = 1.9 dB). The ISI between stimuli ranged from 545 to 609 ms (stimulus onset asynchrony, SOA = 900 ms) for the short ISI condition and 2645–2709 ms (SOA = 3000 m) for the long ISI condition. The entire experiment lasted 2–2.5 h for the short ISI experiment, and 3–3.5 h for the long ISI experiment. Breaks were given halfway through the ERP experiment and whenever the participant requested.

### ERP Recording and Offline Processing

The continuous EEG was time-locked to the onset of the stimuli. The EEG was recorded with a band pass of 0.1–100 Hz, and a sampling rate of 500 Hz from 64 scalp sites using a Geodesic sensor net with the vertex electrode (Cz) as the reference. For offline processing, the EEG was refiltered using a finite impulse response (FIR) band-pass filter of 0.3–15 Hz, and was segmented into 1000 ms epochs, including a 200 ms pre-stimulus baseline. Eye movement artifacts were removed using automatic EOG artifact and eye movement artifact correction via Brain Electrical Source Analyses (BESA) (BESA research 5.2, BESA GmbH, Germany). Epochs that exceeded the amplitude threshold of 120 μV were excluded. After artifact removal, on average, 178.4 trials (89%; *SD* = 15.7) for the /gipa/ deviant condition, 177.8 trials (89%; *SD* = 16.8) for the /gypa/ deviant condition and 408.3 (92%, *SD* = 35.8) trials for the /gupa/ standard condition were included in each individual average. Bad channels were interpolated using the BESA spline interpolation method. The data were re-referenced using the average of all 65 sites and baseline corrected.

## Data Analysis

### “Composite” FzCz Measures

**FIGURE 2:**
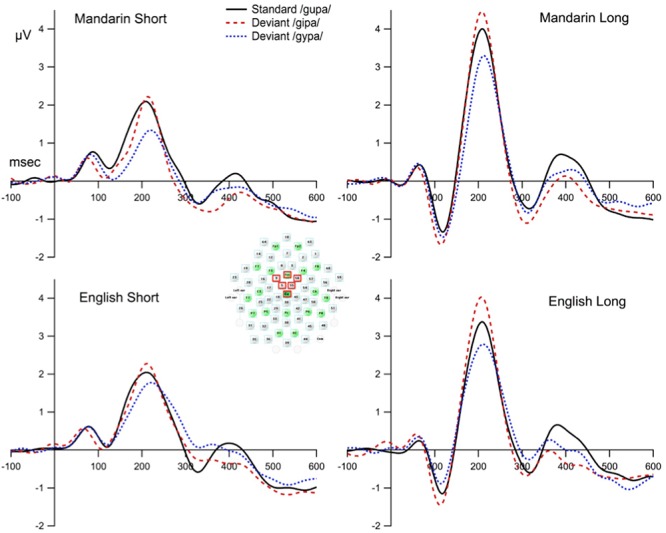
The waveforms at the composite FzCz site for the standard and deviant stimulus condition. The composite FzCz site was generated using the average of six sites near FzCz (these sites are marked with red squares).

To reduce inter-subject variation in the topography of the ERP to speech (Zevin et al., 2010), and to reduce the contribution of independent noise sources at each electrode site to the signal of interest, we built a model of frontocentral activity from six sites around Fz and Cz as follows. We chose Fz and Cz as pivotal sites because MMN is known to have a frontocentral topography, and visual inspection of the data shows that Fz and Cz do indeed have the consistently largest MMN amplitude across participants. We then selected four sites that had the highest average correlation across conditions with Fz and Cz (mean Pearson’s correlation coefficients = 0.92, *SD* = 0.06). Thus, the six sites used in the model were Fz/4, 5, 9, 55, 58, and Cz/65. The average responses from these six sites were treated as one electrode “FzCz composite,” and were used for subsequent statistical analyses. The electrode placement is shown in **Figure 2**.

### ERP Analyses

The subtraction waves were derived by subtracting the ERP waveform evoked by the standard stimuli from the ERP evoked by the deviant stimuli. We downsampled the data from the recording sampling rate of 500–25 Hz using appropriate smoothing to preclude aliasing in Igor Pro, 2017 (Wavemetrics). After downsampling, each data point represented the amplitude for a 40-ms time window. Visual examination of the individual and group average data indicates that the time range for MMN is between 80 and 240 ms, with the /gipa/ deviant showing earlier amplitude peak than the /gypa/ deviant condition. The time range for P3a was between 160 and 360 ms, and the time range for LN was between 340 and 500 ms. We identified the maximal amplitude from the downsampled individual waveforms (the most negative value among the four intervals of 40-ms time windows for MMN and LN, and the most positive value among the five intervals of 40-ms for P3a) from each participant for each component. To examine the presence and absence of MMN, P3a, and LN, we conducted one-sample *t*-tests (one-tailed) to examine whether the maximal amplitude in the 40-ms interval for MMN, P3a, and LN were significantly different from zero.

In a second set of analyses designed to test the language group, ISI condition and deviant type differences in the time range of interest, we used the maximal amplitude of the subtraction waveforms as described above in mixed model ANOVAs with language group (English and Mandarin) and ISI (short and long) as the between-group variable, and deviant stimulus type (gipa and gypa) as the within-group independent invariable.

### Behavioral Analysis

For both the discrimination and identification task, we applied mixed model ANOVAs using language and ISI as the between-group variable, and vowel type as within-subject variable. The response accuracy was the dependent variable.

The Greenhouse–Geisser correction was applied whenever the degree of freedom in the denominator was larger than one. Uncorrected degrees of freedom, corrected *p*-values and generalized eta-squared (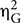) effect size were reported (Olejnik and Algina, 2003). Two-way interactions were examined using Tukeys’ HSD *post hoc* tests, and Bonferroni correction used for multiple comparisons. All the analyses were conducted using R (R Core Team, 2016) and the nlme package (version 3.1-128) (RStudio Team, 2016; Pinheiro et al., 2017).

## Results

### ERP Results

**FIGURE 3:**
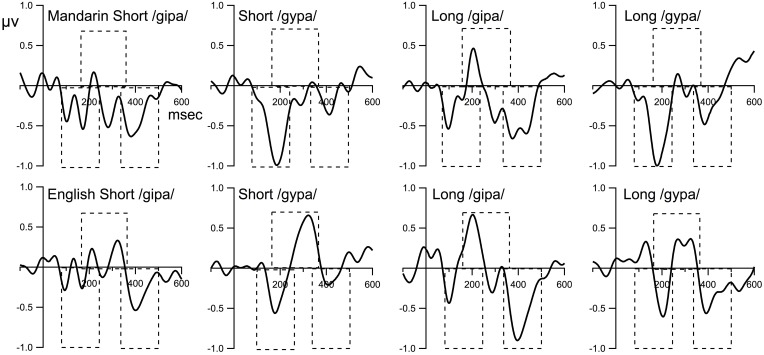
The difference waveforms (deviant minus standard). The time windows for MMN, P3a, and LN are 80–240 ms, 160–360 ms, and 340–500 ms, respectively. Dashed boxes indicate time window used for statistical analysis.

**Figure 2** displays the grand mean ERPs to the standard and deviant stimuli waveforms for the composite FzCz site. **Figure 3** shows the difference waveforms (waveforms from the deviant ERP minus the standard ERP).

#### Presence/Absence of MMN, P3a, and LN

One sample *t*-tests on individual language/ISI groups for each deviant condition showed that the amplitude of MMN for the 40-ms interval that contained the peak was significantly more negative than zero (*p*s < 0.01) for all language/ISI groups under both deviant conditions. In other words, MMN was present in all language/ISI groups. P3a was present for both groups and both deviant conditions, except for the Mandarin short ISI group; specifically P3a was absent for the /gipa/ and the /gypa/ condition for the Mandarin short ISI group. LN was present in all conditions but the English short ISI group for the /gypa/ deviant condition. See **Table 2** for the amplitudes and results from the *t*-tests for each component.

**Table 2:** Means (SD) of the average amplitude for the peak 40-ms interval and *t*-test (one-tailed) results of MMN, P3a and LN components for the four language/ISI groups under /gipa/ deviant and /gypa/ deviant conditions.

		MMN		P3a		LN	
	N	Amplitude (μV)	*t*-value	Amplitude (μV)	*t*-value	Amplitude (μV)	*t*-value
**Deviant = /gipa/**							
Mand Short	16	-0.70 (0.46)^∗∗∗^	-6.11	0.18 (0.61) n.s.	1.27	-0.85 (0.42)^∗∗∗^	-8.08
Mand Long	16	-0.65 (0.47)^∗∗∗^	-5.53	0.65 (0.64)^∗∗^	2.79	-0.87 (0.81)^∗∗∗^	-4.32
Eng Short	16	-0.63 (0.55)^∗∗∗^	-4.6	0.60 (0.61)^∗∗∗^	4.86	-0.62 (0.45)^∗∗∗^	-5.51
Eng Long	15	-0.45 (0.47)^∗∗^	-3.74	0.88 (0.74)^∗∗∗^	5.06	-0.99 (0.54)^∗∗∗^	-7.04
**Deviant = /gypa/**							
Mand Short	16	-1.09 (0.62)^∗∗∗^	-6.99	0.13 (0.61) n.s.	0.90	-0.45 (0.66)^∗∗^	-2.75
Mand Long	16	-1.03 (0.62)^∗∗∗^	-6.60	0.38 (0.64)^∗^	2.37	-0.54 (0.87)^∗∗^	-2.51
Eng Short	16	-0.67 (0.43)^∗∗∗^	-6.22	0.76 (0.61)^∗∗∗^	4.93	-0.24 (0.56)^∗^	-1.73
Eng Long	15	-0.60 (0.63)^∗∗∗^	-3.67	0.59 (0.74)^∗∗^	3.06	-0.62 (0.64)^∗∗∗^	-3.72

**“Mand Long”, Mandarin long ISI condition; “Mand Short”, Mandarin short ISI condition; “Eng Long”, English Long ISI condition; “Eng Short”, English short ISI condition*. *^∗∗∗^p ≤ 0.001, ^∗∗^p ≤ 0.01, ^∗^p ≤ 0.05, ^*n.s*^p-value is not significant.**

#### Language, ISI and Deviant Type Effect on the Amplitude of the Mismatch Negativity

Mixed model ANOVA on MMN amplitude showed a main effect of language [*F*(1,59) = 6.138, *p* = 0.02, 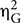 = 0.07] with Mandarin showing larger MMN than the English groups, a main effect of stimulus [*F*(1,59) = 9.99, *p* = 0.002, 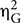 = 0.05] with the MMN amplitude for /gypa/ larger than that for /gipa/. No effect of ISI or interactions with ISI were observed. An interaction of language by stimulus [*F*(1.59) = 3.697, *p* = 0.06, 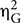 = 0.02] approached significance. We had specifically predicted that English speakers would show poorer discrimination for /gypa/ vs. /gupa/ than for /gipa/ vs. /gupa/, and, thus, followed this with *post hoc* tests. These revealed that the two language groups differed significantly only in the /gypa/ condition with larger MMN for the Mandarin than for the English /gypa/ deviant condition (/gipa/ condition: English mean = -0.54, Mandarin mean = -0.67, *t* = 1.066, df = 59.8, *p* = 0.29; (/gypa/ condition: English mean = -0.63, Mandarin mean = -1.06, *t* = 2.936, df = 60.2, *p* = 0.005).

#### Language, ISI and Deviant Type Effect on the Peak Amplitude of the P3a Responses

The results of mixed model ANOVA revealed a significant main effect of language [*F*(1,59) = 7.620, *p* < 0.01, 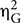 = 0.07] with the English participants showing larger P3a amplitudes. No other main effects or interactions were significant.

#### Language, ISI and Deviant Type Effect on the Amplitude of the Late Negativity (LN)

A mixed model ANOVA on LN amplitude revealed that the only statistical significance was for stimulus [*F*(1,59) = 19.5, *p* < 0.001, 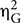 = 0.08], with larger LN to /gipa/ than to /gypa/. No main effects or interactions involved language or ISI.

### Behavioral Discrimination Results

The results for the mixed model ANOVA revealed a main effect of language [*F*(1,59) = 15.9, *p* < 0.001, 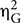 = 0.16] with Mandarin listeners performing better, a main effect of ISI [*F*(1,59) = 16.5, *p* < 0.001, 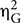 = 0.16] with overall higher performance in the short ISI conditions, and a main effect of stimulus condition [*F*(1,59) = 77.5, *p* < 0.001, 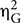 = 0.29] with higher performance in the /gipa/ deviant condition than in the /gypa/ deviant condition. Significant interactions included language by ISI [*F*(1,59) = 8.42, *p* < 0.01, 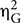 = 0.09], language by stimulus condition [*F*(1,59) = 54.7, *p* < 0.001, 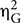 = 0.22], ISI by stimulus condition [*F*(1,59) = 12.5, *p* < 0.001, 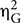 = 0.06], and a three-way interaction of language by ISI by stimulus condition [*F*(2,118) = 16.8, *p* < 0.001, 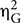 = 0.08]. *Post hoc* tests found that there was no ISI effect in the Mandarin groups; but for the English listeners, the discrimination accuracy was higher in the short than the long ISI condition. Likewise, the Mandarin listeners performed with similar accuracy under /gupa/-/gipa/ and /gupa/-/gypa/ conditions, but the English listeners had lower accuracy in the /gypa/-/gupa/ condition than in the /gipa/-/gupa/ condition. Under the /gupa/-/gipa/ condition, accuracy in the long ISI condition did not differ from accuracy in the short ISI condition, but under the /gupa/-/gypa/ condition, accuracy in the long ISI was lower than in the short ISI condition. See **Table 3** for details.

**Table 3:** Means (SD) of vowel discrimination accuracy and vowel identification under four language/interstimulus (ISI) groups.

	Discrimination		Identification		
	/gipa/-/gupa/	/gypa/-/gupa/	/gipa/	/gypa/	/gupa/
English	0.94 *(0.24)*	0.65 *(0.48)*	0.68 *(0.25)*	0.51 *(0.32)*	0.84 *(0.25)*
Long ISI	0.89 *(0.31)*	0.475 *(0.50)*	0.68 *(0.32)*	0.5 *(0.32)*	0.84 *(0.26)*
Short ISI	0.98 *(0.15)*	0.80 *(0.40)*	0.67 *(0.17)*	0.52 *(0.31)*	0.84 *(0.23)*
Mandarin	0.93 *(0.25)*	0.89 *(0.31)*	0.85 *(0.14)*	0.85 *(0.24)*	0.96 *(0.13)*
Long ISI	0.91 *(0.29)*	0.88 *(0.33)*	0.87 *(0.11)*	0.91 *(0.17)*	0.98 *(0.03)*
Short ISI	0.95 *(0.21)*	0.91 *(0.30)*	0.83 *(0.17)*	0.79 *(0.31)*	0.92 *(0.22)*

*The standard is /gupa/, and the deviants are /gipa/ and /gypa/.*

### Behavioral Identification Results

All groups labeled each category as intended, which was well-above chance level (>33.3%, see **Table 3**). Results from repeated model ANOVA revealed a main effect of language [*F*(1,59) = 30.3, *p* < 0.001, 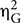 = 0.19] with Mandarin listeners showing higher accuracy. In addition there was a main effect of stimulus [*F*(2,118) = 21.4, *p* < 0.001, 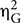 = 0.17]. *Post hoc* tests suggested that the accuracy for both /gipa/ and /gypa/ tokens were significantly lower than for /gupa/ tokens (*p*s < 0.001), and /gypa/ was lower than /gipa/ (*p* = 0.02). A language by condition interaction was also significant [*F*(2,118) = 7.16, *p* = 0.001, 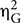 = 0.06], and *post hoc* tests showed that for participants with an English background, /gypa/ was identified with significantly lower accuracy than /gipa/ (*p* < 0.001). However, for Mandarin participants, the accuracy for /gypa/ did not differ from that of /gipa/ (*p* = 0.86). ISI was not a significant factor for identification accuracy.

## Discussion

The aims of this study were to examine how ISI influenced neural responses to infrequent vowel changes, and to what extent language experience modulates the ability to detect speech contrasts. Results demonstrated that ISI and language experience both modulated behavioral performance, but only language experience, and not ISI, modulated the neural response. This is likely due to greater sensitivity of ERP measures to capture temporal dynamics contributing to the behavioral response.

Behavioral results were consistent with the previous literature on cross-linguistic speech perception (e.g., Werker and Logan, 1985; Levy and Strange, 2008). In addition, our hypothesis that language experience modulates the rate of memory trace decay was supported by the behavioral results. Specifically, the English listeners showed poorer discrimination and identification for the difficult /gypa/ vs. /gupa/ contrast and performance was the worst in the long ISI condition. In contrast, ISI did not affect the Mandarin listeners’ behavioral performance.

For ERP results, larger MMNs were observed to the vowel contrasts for the Mandarin speakers than for the English speakers. This finding is consistent with the previous MMN literature on cross-language speech processing (e.g., Dehaene-Lambertz, 1997; Winkler et al., 1999a). We expected the language group difference to be greater for the /gypa/ than the /gipa/ deviant condition, but this interaction only approached significance. In addition, the MMN amplitude was generally larger for the /gypa/ condition than for the /gipa/ condition, but the LN was larger for the /gipa/ than the /gypa/ condition. We did not make specific predications regarding which speech sound contrast would show a greater difference for the Mandarin listeners, but we did expect English listeners to show a larger MMN to the /gipa/ than to the /gypa/ deviant, which we did not observe. P3a was generally larger in the native English speakers than in the Mandarin speakers. This finding supports that English listeners could detect the vowel difference. Finally, counter to our prediction, lengthening the ISI from half a second to 2.6 s for these vowel contrasts did not affect the MMN, P3a or LN amplitudes in either the English or the Mandarin listeners.

These findings are in contrast with the findings on lexical tone processing as reported in Yu et al. (2017). Below, we discuss these findings in greater detail.

### Behavioral Responses and Processing Levels

Our behavioral discrimination results supported our hypotheses and were consistent with the previous literature. Specifically, in the long ISI conditions, listeners had to rely on their native phoneme categories for discriminating speech contrasts, and this resulted in poorer performance for English listeners on the vowel stimuli that were not contrastive in English (Pisoni, 1973; Werker and Tees, 1984; Werker and Logan, 1985; Burnham et al., 1996).

Previous studies using behavioral methods have proposed that speech perception may be influenced by several different factors, such as psychoacoustic auditory, language-general phonetic, and language-specific phonemic factors (Pisoni, 1973; Werker and Tees, 1984; Werker and Logan, 1985; Burnham et al., 1996) depending on the rate of sound presentation. Using an AX discrimination task, Werker and Logan (1985) found that when two stimuli were presented with an ISI of 250 and 500 ms, American–English (AE) listeners were able to discriminate two different CV syllable tokens that were within-category for English listeners (/d/), but across category (dental and retroflex stop consonants) for Hindi listeners. At a longer ISI of 1500 ms, poor performance was observed for American (AE) listeners to the cross-category Hindi contrast. But Hindi listeners maintained good categorization performance. Werker and Logan (1985) interpreted these results as evidence of engaging three different levels of perception. They proposed that under conditions of high stimulus uncertainty and memory load, listeners rely on language-specific categories, while in less demanding task conditions (e.g., low memory demand) discrimination and categorization of speech information can be based on language-general phonetic properties. At the shortest ISIs, listeners can discriminate based on slight acoustic differences.

Our behavioral discrimination experiment differs slightly from the AX paradigms used by previous studies (Pisoni, 1973; Werker and Tees, 1984; Werker and Logan, 1985; Burnham et al., 1996). We adopted a modified version of our ERP oddball paradigm (A_1_A_1_A_2_A_1_X or A_1_A_2_A_1_A_1_X) for the purpose of examining correlations between behavioral and neurophysiological responses. Instead of using an ISI 500 ms vs. 1500 ms, we used an SOA of 900 ms vs. 3000 ms (equivalent to ISI of about 575 and 2675 ms, on average). Therefore, our long ISI condition was considerably longer than the ISI of 1500 ms. The rationale for using a longer ISI was based on the result of our pilot studies for the ERP response to lexical tone.

It is possible that an ISI of 2675 ms was inadequate to observe a difference in the ERP responses, suggesting a dissociation between behavioral and neurophysiological measures under certain conditions. The alternative explanation is that the ISI of 575 ms (900 ms SOA) for the short condition was too long to engage acoustic-phonetic discrimination. The previous studies used simple consonant-vowel or vowel stimuli that were relatively short in duration (less than 300 ms). The more complex stimuli in the current study were likely to increase the reliance on phonemic levels of processing (Strange, 2011). Thus, it is possible that a shorter ISI would result in a larger MMN for the English group (but no change for the Mandarin group). Irrespective of this possibility, our results reveal that the both native- and non-native-language groups maintained sufficient information to allow neural discrimination at the long ISI, but that this information did not support good behavioral perception of the /y/ vs. /u/ vowel for the English listeners.

In general, our behavioral discrimination results support the previous findings in that under long ISI conditions, listeners have to rely on their native phonemic categories for discriminating speech contrast. In our study, both the Mandarin and English listeners discriminated the /gipa-gupa/ contrast with similarly high accuracy under both short and long ISI conditions. However, for the /gypa-gupa/ contrast, the English listeners have lower accuracy in the short ISI condition, and more than 25% of the participants were at chance level in the long ISI condition. This pattern suggests that English listeners were able to use some language-general phonetic information for discrimination under the short ISI condition, but could not employ this information at the longer ISI, because the phonetic trace had decayed too much. Thus, the longer ISI condition reveals that English listeners have assimilated the /y/ vowel into the /u/ phonemic category. This finding was predicted and is consistent with Best’s Perceptual Assimilation Model (Best, 1995; Best and Tyler, 2007).

Examination of the identification response patterns showed that /gipa/ tokens were only occasionally “mis”-labeled as “gupa” (4 and 15% of total “non-gipa responses” for the Mandarin and English groups, respectively) by either language group, while labeling /gypa/ tokens as “gupa” accounted for 96% of total “non-gypa” responses for the English listeners; only 57% of the “non-gypa” responses were due to mislabeling /gypa/ tokens as “gupa” in the Mandarin listeners. So the behavioral identification result provide further evidence that American English listeners assimilate Mandarin /y/ to the English /u/ category. All the stimuli were produced by a native Mandarin speaker. The high discrimination and identification accuracy for /gipa/ and /gupa/ suggested that English listeners assimilated these Mandarin vowels into two difference English vowel categories, consistent with the two-category (TC) assimilation pattern in PAM (Best, 1995; Best and Tyler, 2007). Levy and Strange (2008) illustrated that vowel assimilation including the assimilation of front-rounded vowel /y/ into American English categories is dependent on context (i.e., preceding and following phonemes), task (passive vs. active listening, categorization vs. perceptual assimilation tasks) and listener factors (e.g., Levy and Strange, 2008; Strange et al., 2009).

### Duration of Auditory Memory for MMN, P3a, and LN

An MMN to a pure tone deviant can be present under conditions with an ISI up to about 10–30 s, but the MMN was shown to reduce in amplitude as the ISI increased (Mäntysalo and Näätänen, 1987; Böttcher-Gandor and Ullsperger, 1992; Sams et al., 1993; Winkler and Cowan, 1996, 2005), especially in children and in the clinical population (see Bartha-Doering et al., 2015 for a review). These studies provided important data on the basic auditory sensory memory processing mechanism in humans. However, pure auditory tones differ from complex stimuli in a number of ways. In particular, the relevance of pure tone stimuli (when not part of a melody) is quite different from other complex auditory stimuli, such as speech. Our recent study on lexical tone revealed that differences in experience with speech information (native vs. non-native) modulate the time course of sensory memory decay for this information in the system as indexed by MMN (Yu et al., 2017). In the current study, we did not find evidence of ISI modulation in the MMN, P3a or LN amplitudes. Future studies need to examine memory trace decay of other types of complex (e.g., environmental sounds) and/or relevant auditory information (e.g., music) to determine whether the decay rate for well-learned speech categories is comparable to other well-learned auditory categories. In addition, it will be important to determine where the memory trace to the spectral information in vowels (in this case, primarily the first and second formants) decays much faster than for auditory tones, in that our “short” ISI may have been too long for the vowels in our study to observe the decay effect. In other words, the participants already needed to engage a phonological level of processing with an ISI of 575 ms for the natural speech in this study.

The fact that the MMN was present in all groups regardless of ISI and language background supported the earlier MMN literature on vowel processing (Aaltonen et al., 1987, 1994; Dehaene-Lambertz, 1997; Dehaene-Lambertz and Baillet, 1998; Sharma and Dorman, 1999, 2000; Winkler et al., 1999a,b; Shafer et al., 2004; Sussman et al., 2004; see Näätänen et al., 2007 for a review). It also expanded the current literature by showing that the MMN can be elicited for non-native vowels even when the ISI is greater than 2.5 s. This is not the case for other speech categories such as lexical tone or consonant (Čeponienė et al., 1999; Yu et al., 2017). Using the same paradigm, we have found that the MMNs for a lexical tone contrast were absent in the English speaker groups when the ISI was long (Yu et al., 2017).

The absence of MMN amplitude attenuation in the long ISI conditions for either native or non-native vowel contrasts compared to the MMNs in the short ISI conditions suggests that the duration of auditory sensory memory for these non-native vowel contrasts is longer than that for non-native lexical tone contrast (at least for Mandarin tone 2 vs. 3). The alternative, is that auditory sensory memory to vowels is much shorter (and thus, the short and long ISI conditions in this study, both precluded acoustic-phonetic discrimination). Under either interpretation, our findings are consistent with recent behavioral findings that different types of speech are weighted differently in lexical access. The explanation that auditory sensory memory for vowels is longer than for lexical tone contrasts matches well with the findings that vowels are weighted more than consonants and lexical tone (Wiener and Turnbull, 2016).

The current study did not provide direct MMN evidence about the rate of sensory memory decay for vowel contrast, but it does show, for the first time, that the sensory memory for a vowel contrast lasts longer than 2.6 s; this is longer than the 2 s reported for consonants in 7- to 9-year old children with good phonological memory skills, and also longer than that reported for lexical tone in non-native speakers. Future studies should use both longer and shorter ISIs to examine if and how ISI influences the amplitude of MMN for vowel contrast.

The question of whether and how the P3a response changes as a function of ISI has rarely been examined. Friedman et al. (2001) proposed that the P3a is “associated more with the evaluation” of detected deviant events “for subsequent behavioral action.” The findings in this study suggest that this evaluation process, following deviance detection, is not influenced by an ISI difference of 2 s for vowels.

We predicted that P3a amplitude would be smaller under more challenging conditions such as when the magnitude of stimulus deviance is small (e.g., the /gypa/ deviant condition) or when memory decay is greater (e.g., the long ISI condition). However, we did not see evidence of ISI modulation of P3a, nor evidence of a stimulus deviance effect. Instead, we saw an effect of language experience. That is, the English listeners had larger P3a than the Mandarin listeners, suggesting that the English listeners automatically oriented to the deviance more. This finding is consistent with the literature, which has observed larger P3a amplitude for non-native contrasts and for later-learned contrasts (Shestakova et al., 2003; Ortiz-Mantilla et al., 2010). We re-examined the P3a data from the lexical tone study in Yu et al. (2017), which revealed that P3a was also larger in the English groups than the Mandarin groups to lexical tone contrasts. The larger P3a amplitude in the English listeners to the Mandarin or less-prototypical English contrasts may indeed reflect an increased level of “perceptual vigilance,” as suggested by Ortiz-Mantilla et al. (2010). It is possible that the less native-like deviant is not as predictable as the native deviance, especially in a multiple deviance paradigm, since fully predictable deviance leads to absence of P3a and LN (Ritter et al., 1999; Sussman et al., 2003).

We did not find clear evidence that ISI affected the LN amplitude. The LN amplitude differed under the two deviant conditions, with larger LN for the /gipa/ deviant than for the /gypa/ deviant condition. This is the opposite pattern to the MMN responses. As discussed above, the MMN amplitude was larger in the /gypa/ than the /gipa/ deviant condition. The phonetic-phonological properties of /y/, /i/, and /u/ support a greater difference between /i/ an /u/ than between /y/ and /u/ because the /i/ differs from /u/ in both the back and round feature, whereas /y/ only differs in the back feature. Several studies of language/learning differences have found reduced MMN and comparable or larger LN in the less proficient language groups (e.g., family history of language impairment: Addis et al., 2010; children with SLI: Shafer et al., 2005; second language learners: Ortiz-Mantilla et al., 2010). Some studies also found the opposite with smaller LN in the less proficient language users/learners (Barry et al., 2009; Bishop et al., 2010). Therefore, the nature of LN still requires explanation. It seems that, at this time, more evidence supports the account that the LN reflects additional recruitment of cognitive resources for further processing of the sound contrast, as it can be generated independently of the amplitude of MMN. Across studies, LN is sometimes absent when MMN is robust, especially when the task is easy, such as in this study. LN was present but very small in the short ISI /gypa/ condition, in which large MMNs were present.

One neuronal network operation principle is to minimize the cost (Bullmore and Sporns, 2012). Based on this principle, it is feasible to propose that MMN and LN represent a two-stage sequential process. If the processing is sufficient and adequately automatic during the early processing time window as indexed by a robust MMN, then no further processing is necessary, thus no LN will be elicited. For a difficult vowel contrast or challenging perceptual condition, the early automatic discrimination may or may not take place, and LN represents the recruitment of additional resources. We do not see a clear association between P3a and LN amplitude. However, we did not design our study to examine the association/disassociation of MMN, P3a and LN. The earlier three-stage sequential model (MMN-P3a-LN, or MMN-P3a-RON) was proposed by Escera and Corral (2003, 2007) and other colleagues. Our study supports the pair-wise dissociation of the three ERP components suggested by Horváth et al. (2008b). The rate of sensory memory decay does not change the pair-wise dissociation of MMN, P3a and LN amplitude.

### The Dissociation Between Behavioral and Neurophysiological Responses

In the behavioral literature, an ISI of 1500 ms was adequate to lead to lower discrimination accuracy to consonant contrasts; however, in the ERP literature, the MMN amplitude to a pure tone contrast can be robust even when the ISI was beyond 10 s. We were interested in whether the linguistic nature of the stimuli would make a difference to the ISI modulation effect. In the lexical tone study, we found that an ISI of 2.5 s led to MMN and LN absence/reduction and lower behavioral discrimination accuracy. However, in this study of vowel deviance, we found a dissociation between the behavioral discrimination accuracy and neurophysiological responses. Our findings for behavioral discrimination support the prediction that a longer ISI leads to more reliance on phonemic-level processing. In contrast, the lack of an ISI effect on the MMN, P3a, and LN amplitude for either language group suggests that phonetic level information is still available for vowel contrasts up to an ISI of 2.5 s. The dissociation between behavioral and neural measures has been reported previously (e.g., Shafer et al., 2005). Discrimination accuracy rate can be influenced by many other factors such as focused attention, inhibition, and working memory that are less apparent in the passive listening paradigm. The association and dissociation between behavioral and neural measures needs to be further examined more systematically in future studies.

### Vowel vs. Lexical Tone Processing

Overall, we found that the neural responses to the vowel contrasts differed from the lexical tone contrasts, although the behavioral pattern of responses to the vowel contrasts were very similar to that of the lexical tone contrast. These neural discrimination differences may indicate functional and origin variations at the cortex level (e.g., Shestakova et al., 2004; Bouchard and Chang, 2014).

We have evidence of ‘tonotopic’ maps in the auditory cortex from early MEG studies (Romani et al., 1982; Pantev et al., 1988; Shestakova et al., 2004; Talavage et al., 2004). As shown in Shestakova et al. (2004), the amplitudes and source location of N1 differs for different vowel (e.g., Russian vowels [a], [i], and [u] used in the study), and vowels with more dissimilar spectral envelopes are more distantly coded at the cortex level. Recent electrocorticographic recordings suggested that the ventral sensory-motor cortex (vSMC) is the origin of neural activity that exerts precise vocal tract movements (Bouchard and Chang, 2014). The same research group has also reported that posterior superior temporal gyri (pSTG) serve as a critical locus for voice-onset time in consonant production (Chang et al., 2010). The majority of human imaging studies suggested that the lateral end of Heschl’s sulcus, anterolateral to primary auditory cortex, is the center for processing fundamental frequency (Patterson et al., 2002; Penagos et al., 2004; Wang, 2013). Thus, ERP differences among vowel, consonant and lexical tone units could reflect different neural origins and functions at the cortical level. It is possible that differences in sensory memory decay effects for lexical tone vs. vowel contrast (/gypa/-/gupa/) is related to these functional differences. However, this difference could be due to acoustic salience. It is challenging to equate acoustic difference across different acoustic properties (e.g., stimulus duration, fundamental frequency, spectral information in formants), which complicates explaining these differences. Future studies that manipulate the degree of difference within and across these properties are necessary to fully understand how these relate to neural discrimination and behavior.

### Stimulus Variable and the MMN Latency of /gipa/ Deviant

Many cross-language studies have used synthetic speech to allow strict control over the variance of acoustic parameters. However, natural speech produced by human speakers is quite variable, and the multiple acoustic parameters that are exploited by native listeners to differentiate the phonemic categories are still not entirely understood. The perceptual patterns observed for highly controlled synthetic speech may not reflect the reality of everyday speech perception. Thus, a more ecologically valid task, as promoted by Strange and Shafer (2008), is the use of natural speech. In the current study, we used two tokens per deviant category, and three tokens for the standard category of naturally produced bisyllabic non-words. These tokens were selected from a large pool of recordings based on careful listening and detailed acoustic analysis of voice onset time for /g/ and /p/, vowel onset and offset time, vowel formant frequencies, F0 contour, overall amplitude and duration, duration and amplitude of each segment within the syllable, and to ensure that phonetically irrelevant acoustic variability was not highly correlated with the phonetically relevant acoustic variability, but to allow for the natural variability found in everyday speech. The syllable durations for the two tokens of /gi/ were approximately 20–25 ms shorter than those of /gu/, and the syllable duration for one token of /gy/ was approximately 18 ms longer than those of /gu/. The coarticulatory cues in the voicing (which was prevoicing) would allow differences of the deviant stimuli to be calculated from stimulus onset. Thus, the earlier MMN to the /gi/ syllable compared to /gy/ syllable cannot be explained by the differences in syllable duration. It is possible that a later MMN, related to syllable duration differences could add into the MMN amplitude. However, it is important to recognize that our goal was not to specifically compare the difficultly of neural discrimination of /i/ and /y/ from /u/. Rather it was to determine the effects of language experience and short-term memory decay on neural discrimination of highly natural vowels.

By implementing such natural speech, we hoped to tap into phonemic processing to a greater extent than found in processing synthetic speech without introducing so much variability that the “noise” masked the contrast of interest. The behavioral responses indicate that use of natural speech is adequate. Both deviant types /gi/ and /gy/ are illegal syllables in Mandarin (/gu/, /ga/, and /ge/ are allowed), and /gipa/, /gypa/, and /gupa/ are all non-words in both Mandarin and English. Therefore, phonotactic probabilities (biphone probability) were similar for the two language groups.

One unexpected finding was the latency of the MMN for /gipa/ deviant appears to peak quite early at around 100 ms, but its amplitude is smaller than that of /gypa/ regardless of ISI and language groups. It is established that an easy contrast will lead to earlier MMN latency. The acoustic difference in terms of all the parameters mentioned in the paragraph above for /gipa/ and /gypa/ are quite subtle except for in F2 and F3. For these values, /gipa/ is acoustically more distinct than /gypa/ from /gupa/, in part due to the round-feature difference. This greater difference is consistent with an earlier MMN. Separating the behavioral and ERP data by deviant tokens suggests that one token of /gipa/ (Token 2 in **Table 1**) was identified with lower overall accuracy (75% for Token 2 vs. 95% for Token 1) than the other token by the 32 Mandarin listeners, and the MMN for this token was later in the Mandarin short ISI group. This may partly account for the two peaks for /gipa/ and the one for the /gypa/ deviant. Regardless of the explanation, our main interest was in the group difference. The fact that both groups showed these patterns, suggest that the acoustic-phonetic level of processing is the source of this difference.

## Conclusion

To our knowledge, this is the first neurophysiological study examining the role of long-term memory representations for vowel processing. We found that behavioral discrimination accuracy was reduced in the long ISI compared to short-ISI condition for non-native vowel deviance. In contrast, no amplitude differences were observed for MMN, P3a, and LN between the short- and long-ISI memory trace decay conditions. For both ISIs, MMN was elicited, and it was larger for the Mandarin than English group, particularly for the difficult Mandarin contrast. This finding suggested that echoic sensory memory trace maintenance for vowels differs from that for lexical tone (fundamental frequency). Our future goals are to evaluate specific hypotheses concerning mechanisms underlying MMN dysfunction in memory disorders such as schizophrenia, children with phonological working memory deficit, or low language proficiency.

## Author Contributions

YY and VS: developed the paradigm. YY, VS, and ES: data analysis, manuscript writing.

## Conflict of Interest Statement

The authors declare that the research was conducted in the absence of any commercial or financial relationships that could be construed as a potential conflict of interest.
